# The effect of wuling capsule on depression in Type 2 diabetic patients

**DOI:** 10.1042/BSR20191260

**Published:** 2020-02-18

**Authors:** Huanping Wang, Huizhen Chen, Yang Gao, Shengju Wang, Xian Wang, Xiaomei Tang, Wei Fang, Xiaoyan Shi, Jia Yao, Qiu Chen

**Affiliations:** Department of Endocrinology, Hospital of Chengdu University of Traditional Chinese Medicine, China

**Keywords:** Depression, Inflammatory factors, Oxidative stress, Type 2 diabetes mellitus, Wuling capsule

## Abstract

**Objective:** Depression is a common complication in Type 2 diabetes mellitus (T2DM); however, it has long been underrecognized and undertreated. In the present study, we aimed to evaluate the clinical effects of Wuling capsule, a compound traditional Chinese herbal medicine, on T2DM complicated with depression.

**Method:** 66 patients were enrolled and randomly divided into Wuling capsule treatment group and placebo group, and finally 64 cases finished the present study. The levels of FPG, 2hPG, HbAlc, TNF-α, IL-6, SOD, MDA, Cor, ACTH, HOMA-β, HOMA-IR and ISI of patients were evaluated and compared. The HAMD scale for patients were recorded.

**Result:** After 12-week treatment, the HAMD scale decreased in both groups, and was lower in Wuling capsule group. The level of FPG in Wuling capsule group was significantly lower than in placebo group; however, no obvious changes of 2hPG and HbA1c were found. The levels of IL-6 and TNF-α were significantly decreased in both groups, and more obviously in Wuling capsule group. The level of SOD was increased while the level of MDA was decreased significantly in both groups, and the changes were more obviously in Wuling capsule group. The levels of Cor and ACTH were significantly decreased in both groups; however, there was no statistically significance between the two groups. Besides, the comparisons of HOMA-β, HOMA-IR and ISI between the two groups were not statistically significant.

**Conclusion:** Our results suggested that Wuling capsule ameliorated the depression in patients with T2DM, and also improved the state of inflammation and oxidative stress state. These results also strongly indicated the ability of clinical transformation of Wuling capsule in patients with T2DM in the future.

## Introduction

Previous studies have found that 64% patients with diabetics were associated with different levels of psychological distress, and finally 24% patients with diabetics were diagnosed with depression [1]. Depression increases the risk in developing Type 2 diabetes mellitus (T2DM) and the subsequent risks of hyperglycemia, insulin resistance, and micro- and macrovascular complications. On the other hand, T2DM also increases the incidence of depression [2]. Therefore, the combination of T2DM and depression seriously comprises long-term prognosis and the life quality of the patients [3]. Thus, it is very important and necessary to effectively treat depressive and anxiety symptoms in patients with T2DM.

Drug treatment and psychological treatment are preferred methods to improve the mental state and alleviate depressive symptoms. Clinically used antidepressant drugs include monoamine oxidase inhibitors, tricyclic antidepressants and selective 5-serotonin reuptake inhibitors (SSRIs). The treatment options are largely depended on the consideration of side effects, patient preferences and individual responses. The application of anti-depressive medication has been practically restricted due to the inevitable side effects, especially the blood glucose increase, weight gain and hyperglycemia [4]. Therefore, the medicines for depression and T2DM should be explored further.

In traditional Chinese medicine, Wuling capsule is isolated from natural Radix Anophylla. It belongs to the subphylum of ascomycetes, Xylariales, Xylariaceae. C. nigra (chicken fir eggs, Poria cocos, Wulingshen in Chinese folk name), a crude drug collected in Sichuan Journal of Traditional Chinese Medicine [5] and a Chinese herb for supplementing qi in Sichuan, Yunnan, Guangdong and other places [6]. Previous researches have showed that Wuling capsule had antianxiety and antidepressant effects in post-stroke patients [7]. Also, researchers have found that it had the function of controlling blood sugar [8]. Hu Yuankui’s study showed that it had certain effect on the improvement of islet function [9]. Collectively, these studies suggest that Wuling capsule has both antidepressive and decreasing blood glucose effects.

In the present study, we will evaluate the clinical effect of Wuling capsule in T2DM patients complicated with depression through a randomized double-blind experiment. We will also observe the effects of Wuling capsule in inflammatory factors, oxidative stress level, HPA axis function and pancreatic islet function, thus providing the data support for its large-scale clinical application in the future.

## Methods

### Subjects

The subjects were enrolled from the Department of Endocrinology of Affiliated Hospital of Chengdu University of Traditional Chinese Medicine, who met the inclusion criteria of T2DM. The present study was approved by the ethics committee of Chengdu University of Traditional Chinese Medicine. Written informed consent has been obtained from all subjects. The research was conducted ethically in accordance with the World Medical Association Declaration of Helsinki. The detailed inclusion and exclusion criteria for the present study were listed in Table 1.

**Table 1:** The detailed inclusion and exclusion criteria for the present study

Inclusion criteria
**(1)** Patients diagnosed with Type 2 diabetes (following the WHO diabetes diagnosis and typing standard (1999) diagnostic criteria).
**(2)** Moderate and mild depression (light depression), depression and recurrent depression with non-psychotic symptoms (light depression, depression with non-psychotic symptoms, following diagnostic criteria of 10th edition of 1992 WHO International Classification of diseases – mental and behavioral disorders).
**(3)** Score more than 8 points, less than 35 points according to Hamilton Depression Scale (HAMD, 24 versions); accord with the diagnostic criteria of imbalance of heart and kidney in traditional Chinese medicine.
**(4)** Adult patient over 18 years of age.
**(5)** Glycated hemoglobin was less than or equal to 8%.
**(6)** No antidepressants and psychotropic drugs were used within 2 weeks.
**(7)** The patient had self-knowledge ability and correctly understood the questionnaire content, and written informed consent was obtained from the patients before the study

Exclusion criteria
**(1)** Patients with acute complications of diabetes, including diabetic ketoacidosis, diabetic hyperosmolar nonketotic coma, hypoglycemia coma or severe unconscious hypoglycemia, etc.; or severe chronic diabetic complications.
**(2)** Patients with heart failure, unstable angina pectoris, severe arrhythmia, myocardial infarction in the past 12 months; blood pressure SBP>180mmHg or DBP>100mmHg.
**(3)** Patients with a history of liver disease such as cirrhosis, hepatitis B, or hepatitis C (except for carriers), or AST or ALT 2. 5 times higher than the normal upper limit.
**(4)** Patients with renal disease or clinically diagnosed history of renal insufficiency, serum creatinine greater than 1.5 mg/dl (132.6 mol/l) and the clearance rate of endogenous creatinine less than 50 ml/min.
**(5)** Patients who suffered from acute or chronic pancreatitis, or had pancreatic injury for any reason, had blood amylase levels above the normal limit.
**(6)** Patients were suffering from upper gastrointestinal ulcers, history of bleeding and gastric retention due to gastrointestinal surgery or pyloric obstruction.
**(7)** Patients with other endocrine system diseases, such as hyperthyroidism, hypercortisolism and so on.
**(8)** Patients with severe mental symptoms other than depression or family history of mental disorders and mental disorders of family history.
**(9)** There were stress factors such as trauma, infection, surgery, fever and history of glucocorticoid use in the last 3 months.
**(10)** No clear history and typical clinical manifestations of HPA axis dysfunction (centripetal obesity, full moon face, buffalo back, skin purple pattern, etc.)
**(11)** Pregnant women, breast-feeding women, women of childbearing age who did not take effective contraception or plan to conceive during the trial period, whose results of urine HCG test show positive.
**(12)** Patients with perimenopausal syndrome.
**(13)** Patients with that curative effects and safety judgment was affected by data under-collection or unable to judge.

### Grouping

According to the inclusion and exclusion criteria, 66 cases with T2DM combined with depression were randomly assigned into two groups: Wuling capsule group (basic therapy + Wuling capsule, 33 Cases) and placebo group (basic therapy + placebo, 33 Cases). The tablets of Wuling capsules or placebo (Zhejiang zuoli Pharmaceutical Co. Ltd.) were taken orally three times a day after meal. The basic therapy consisted: (1) Sulfonylurea drugs: Glimepiride 1–2 mg, qd; Regellinide 1 mg, tid; Metformin 0.75–1.7 g, qd; Pioglitazone 15–30 mg, qd. (2) Insulin or insulin analogues. (3) Statins: Simvastatin 40 mg, qd; Atovastatin 20 mg, qd. (4) Antihypertensive drugs: Captopril l2.5 mg, tid; Valsartan 80 mg, qd; Losartan 50 mg, qd; Nifedipine controlled-release tablet 30 mg, qd; Metoprolol 25–50 mg, bid; Bisoprolol 2.5 mg, qd; Furosemide 20–40 mg, qd. The medical history for patients in the two groups was listed in Table 2. The dosage of hypoglycemic drugs except acarbose and the blood glucose level of the two groups were controlled in the same way. The intervention time was 12 weeks.

**Table 2:** Demographic and baseline characteristics in two groups of patients (means ± s)[M (QR)]

Characteristic	Wuling capsule group	Placebo group	*P* value
Female	16	18	0.441
Age (year)	59 ± 3	58 ± 2	0.342
BMI (kg/m^2^)	23.48 ± 4.14	23.64 ± 2.61	0.854
Medical history			
Hyperlipidemia	26	24	0.873
Hypertension	28	30	0.970
HbA1c (%)	6.95 ± 1.00	7.02 ± 0.94	0.749
FPG (mmol/l)	8.92 ± 2.13	9.05 ± 2.01	0.802
2hPG (mmol/l)	10.41 ± 2.90	10.30 ± 2.48	0.855
HAMD	17.65 ± 5.30	16.88 ± 5.82	0.577
IL-6 (pg/ml)	9.24 ± 1.92	8.62 ± 1.74	0.187
TNF-α (ng/ml)	17.75 ± 3.86	16.18 ± 4.52	0.141
SOD (U/ml)	93.37 ± 10.75	89.33 ± 17.70	0.274
MDA (ng/ml)	377.44 ± 64.27	388.17 ± 55.94	0.479
Cor (µg/l)	220.43 ± 63.01	224.29 ± 61.81	0.806
ACTH (pg/l)	296.93 ± 57.39	303.53 ± 67.08	0.674
HOMA-IR**^A^**	2.90(2.59)	3.23(1.59)	0.340
HOMA-β**^A^**	35.12(50.37)	30.54(27.11)	0.238
ISI**^A^**	0.015(0.010)	0.013(0.007)	0.294

### Observational index

To evaluate the effectiveness of Wuling capsule, the following indexes were compared intragroup and intergroup before and after 12-weeks treatment. The patients’ venous blood was measured at 0 weeks and 12 weeks after treatment, and the levels of blood glucose (fasting plasma glucose, FPG; 2-h postprandial glucose, 2hPG), glycated hemoglobin A1c (HbA1c), tumor necrosis factor α (TNF-α), interleukin 6 (IL-6), superoxide dismutase (SOD), malondialdehyde (MDA), cortisol (Cor) and adreno-cortico-tropic-hormone (ACTH) were detected using the standard clinical laboratory methods. The Hamilton depression scale (HAMD) was assessed by doctors who are blinded to the grouping. The homeostasis model assessment of insulin resistance (HOMA-IR), insulin sensitive index (ISI) and HOMA-β were calculated based on the levels of fasting insulin (FINS, electrochemical luminescence method for insulin detection) and FPG, and the calculation methods were listed below:

① HOMA-IR:HOMA-IR = (FPG × FINS)/22.5;
② ISI:ISI = 1/(FPG × FINS);
③ HOMA-β:HOMA-β = (20 × FINS)/(FPG − 3.5).

### Statistical analyses

The statistical analysis of the data was carried out by SPSS 22.0 software. The quantitative data were shown as the mean ± standard deviation (mean ± SD); Qualitative indicators were described in terms of frequency, percentage or constituent ratio. Paired *T* test was used for comparison within-group. Independent-sample *T* test was used to compare the differences between groups. Nonparametric test was used for abnormal distribution with the Mann–Whitney *U* tests. The enumeration data in the classified data was checked by chi-square test. Grade data were checked by non-parametric test. When HOMA did not conform to normal distribution, statistical analysis was carried on after logarithmic transformation. Statistical description of HOMA was shown as median (quartile spacing). *P* < 0.05 was considered as statistically significant.

## Result

### The demographic and baseline characteristics for patients in each group

After the enrollment, 1 case in treatment group and 1 case in control group terminated the follow-up treatment. Therefore, 64 cases (32 cases in treatment group and 32 cases in control group) finished the study. Before the treatment, we investigated and examined basic indexes for these patients. We found that there was no statistically significant difference in gender, age, BMI, medical history, and the levels of FPG, 2hPG, HbA1c, TNF-α, IL-6, SOD, MDA, Cor, ACTH, HOMA-IRA, HOMA-βA and ISIA between the Wuling capsule group and the placebo group (*P* > 0.05) (Table 2).

### Wuling capsule obviously decreased the levels of FPG, 2hPG and HbA1c

**Figure 1:**
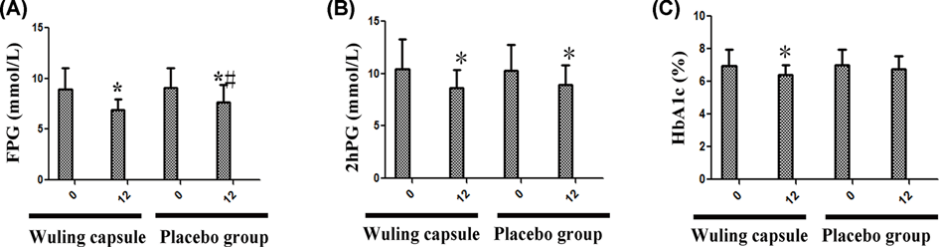
Comparisons of blood glucose and glycosylated hemoglobin in two groups after intervention After 12-weeks intervention, the levels of FPG and 2hPG were significantly decreased in both groups (**A** and **B**). HbA1c only decreased significantly in the Wuling capsule group (**C**). Besides, only the level of FPG in Wuling capsule group was significantly lower than in placebo group (*P* < 0.05) (A). Comparisons before and after intervention within the groups: *P < 0.05; vs Placebo group: ^#^*P* < 0.05.

After 12-weeks intervention trial, the levels of FPG and 2hPG were significantly decreased in both groups (*P* < 0.05) (Figure 1A,B). HbA1c decreased significantly in the Wuling capsule group (*P* < 0.05) (Figure 1C). The levels of FPG in Wuling capsule group were significantly lower than in placebo group (*P* < 0.05) (Figure 1A). The comparison of 2hPG and HbA1c I between the Wuling capsule group and the placebo group was not statistically significant (*P* > 0.05) (Figure 1B,C).

### Wuling capsule ameliorated the HAMD level

**Figure 2:**
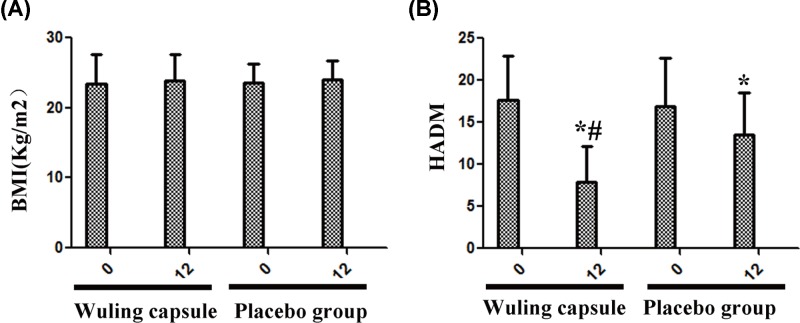
Comparisons of BMI and HAMD between the two groups after intervention After the treatment, the score of HAMD was significantly decreased in both groups, and the score of HAMD in Wuling capsule group was significantly lower than in placebo group (**B**). The BMI of the two groups were on the rise, however the differences were not statistically significant (**A**). Comparisons before and after intervention within the groups: *P < 0.05; vs Placebo group: ^#^*P* < 0.05.

After 12 weeks of treatment, the scores of HAMD were significantly decreased in both groups (*P* < 0.05) (Figure 2B). The BMI of the two groups were on the rise, and the differences were not statistically significant (Figure 2A). The score of HAMD in Wuling capsule group was significantly lower than in placebo group (*P* < 0.05) (Figure 2B).

### Wuling capsule decreased the levels of inflammatory factors IL-6 and TNF-α

**Figure 3:**
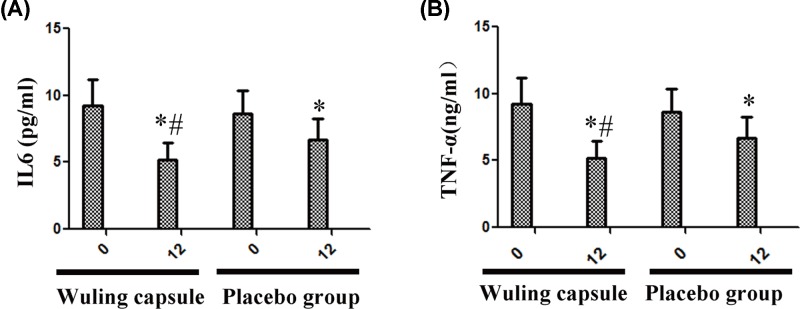
Comparisons of levels of inflammatory factors between the two groups after intervention After 12-weeks intervention, the levels of IL-6 and TNF-α were significantly decreased in both groups (**A** and **B**). Moreover, the levels of IL-6 and TNF-α in Wuling capsule group were significantly lower than those in the placebo group after treatment (A and B). Comparisons before and after intervention within the groups: *P < 0.05; vs Placebo group: ^#^*P* < 0.05.

After 12-weeks intervention, the levels of IL-6 and TNF-α were significantly decreased in both groups (*P* < 0.05) (Figure 3A,B). Besides, the levels of IL-6 and TNF-α in Wuling capsule group were significantly lower than those in the placebo group after treatment (*P* < 0.05) (Figure 3A,B).

### The level of SOD was increased while the level of MDA was decreased in Wuling capsule group

**Figure 4:**
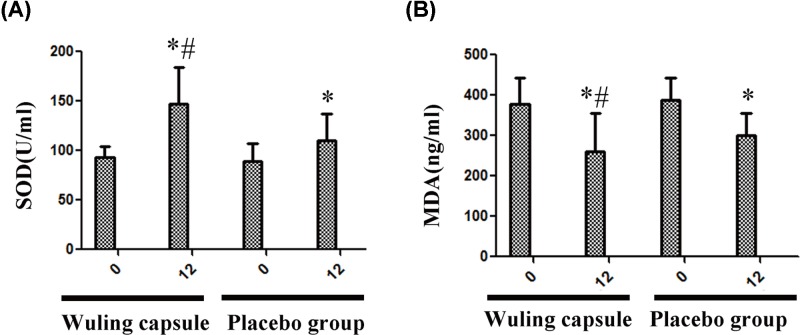
Comparisons of levels of Oxidative stress between the two groups after intervention After 12-weeks intervention, the level of SOD was significantly decreased; however, the level of MDA was increased in both groups (**A** and **B**). Moreover, the level of SOD was significantly higher, and the level of MDA was lower in Wuling capsule group than those in the placebo group after treatment (A and B). Comparisons before and after intervention within the groups: *P < 0.05; vs Placebo group: ^#^*P* < 0.05.

After 12-weeks intervention, the level of SOD was significantly decreased; however, the level of MDA was increased in both groups (*P* < 0.05) (Figure 4A,B). Moreover, the level of SOD was significantly higher, and the level of MDA was lower in Wuling capsule group than those in the placebo group after treatment (*P* < 0.05) (Figure 4A,B).

### The effects of Wuling capsule on the levels of Cor and ACTH

**Figure 5:**
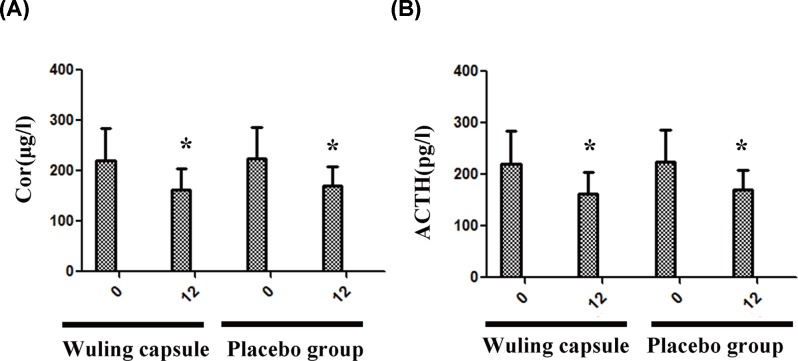
Comparisons of levels of Cor and ACTH between the two groups after intervention After 12-weeks intervention, the levels of Cor and ACTH were significantly decreased in both groups (**A** and **B**). The comparisons of Cor and ACTH between the two groups were not statistically significant (A and B). Comparisons before and after intervention within the groups: *P < 0.05; vs Placebo group: ^#^*P* < 0.05.

After 12-week intervention, the levels of Cor and ACTH were significantly decreased in both groups (*P* < 0.05) (Figure 5A,B). However, the comparisons of Cor and ACTH between the Wuling capsule group and the placebo group were not statistically significant (*P* > 0.05) (Figure 5A,B).

### The effects of Wuling capsule on the levels of HOMA-β, HOMA-IR and ISI

**Figure 6:**
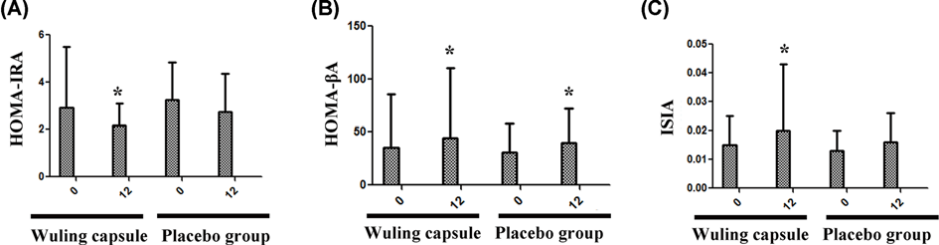
Comparisons of levels of islet function index between the two groups after intervention After 12-week intervention, the level of HOMA-β was significantly increased in both groups (**B**). However, the levels of HOMA-IR and ISI were only improved in Wuling capsule group (**A** and **C**). When compared with placebo group, the levels of HOMA-IRI, HOMA-β and ISI showed no statistically changes (**A–C**). Comparisons before and after intervention within the groups: *P < 0.05; vs Placebo group: ^#^*P* < 0.05.

After 12-week intervention, the level of HOMA-β was significantly increased in both groups (*P* < 0.05) (Figure 6B). However, the levels of HOMA-IR and ISI were only improved in Wuling capsule group (*P* < 0.05) (Figure 6A,C). Compared with the placebo group, the levels of HOMA-IRI, HOMA- β and ISI only showed a trend of improvement after the Wuling capsule treatment, but there was no statistical significance between the groups (*P* > 0.05) (Figure 6A–C).

## Discussion

Depression is one of the common complications in diabetes mellitus, and at the same time, the link between depression and diabetes was intricate. Both the diseases share lots of same pathophysiological pathways and affect the clinical course of each other. In traditional Chinese medicine, Wuling capsule are used to treat anxiety and depression in post-stroke depressive patients [7]. Recent studies showed that it could also control the blood glucose level and improve islet function [8,9]. In the present study, we found that Wuling capsule improved depressive state of patients and had certain auxiliary effect on blood glucose regulation in patients with T2DM combined with depression.

Studies have showed that depression could disrupt the normal blood glucose regulation and increased the risk of diabetic complications [10]. Meta-analysis showed that up to 27. 3% of T2DM patients suffered from depression, which was much higher than in general population [11]. The total score of HAMD can reflect the severity of the depression. Studies showed that Wuling capsule played a role in anti-anxiety through increasing TSPO-mediated mitophagy [1]. Our results showed that the scores of HAMD were significantly decreased in both Wuling capsule group and the placebo group; however, the score of HAMD in Wuling capsule group were significantly lower than in placebo group, indicating that Wuling capsule could obviously improve the depression state of the patients with T2DM.

Previous studies have found that Wuling capsule could significantly reduce the levels of FPG and GA in diabetes patients complicated with insomnia [8]. Hu Yuankui reported that Wuling capsule advanced the peak of insulin secretion and prompted the recovery of β-cell function through a test of 34 patients with Type 2 diabetes after oral administration of Wuling capsule for one month [9]. Our data showed that the levels of FPG, 2hPG and HbA1c were significantly decreased after the 12-week treatment with Wuling capsule. These data indicated that Wuling capsule played an important role in the control of blood glucose level. There were studies found that Wuling capsule promoted the recovery of islet function in diabetic patients [9]. In our study, although we found that there was certain improvement in HOMA-IRI, HOMA-β and ISI after the treatment with Wuling capsule, there was no statistical significance when compared with the placebo group. We speculated this might be related to the small sample size and the relatively short-term intervention.

Researchers believed that inflammation might be the key biological pathway in the development of diabetes and depression. Inflammatory factors can block the cortisol signaling pathway in the body and eventually lead to the severe insulin resistance and glucose metabolism disorder [12,13]. TNF-α is a potent pro-inflammatory factor in diabetic neuropathy. There were no obvious neuropathological changes in TNF-α knockout mice with diabetic [14]. The main cause of morbidity and mortality in patients with diabetes is vascular complications, especially the atherosclerosis. It is believed that the vascular endothelial dysfunction is the early sign of atherosclerosis, and oxidative stress is the main factor causing vascular endothelial dysfunction [15]. Oxidative stress can also induce inflammatory response. Therefore, depression, chronic inflammation and oxidative stress are important risk factors for the prevention and treatment of T2DM. Our results showed that the levels of inflammatory factor (TNF-α and IL-6) and oxidative stress indicators (SOD and MDA) were significantly decreased after the treatment with Wuling capsule.

The HPA axis is a hub of nerve-endocrine network, which includes direct action and feedback interaction between the hypothalamus, pituitary and adrenal glands [16,17]. It regulates physical activity in a quiet state, including digestion, immunity, emotion, learning, sexual behavior, sleep, and energy storage and consumption, and especially plays an important role in the regulation of stress response [18,19]. Diabetes is an endocrine disorder due to insulin deficiency and (or) insulin resistance. It is believed that there is a close relationship between diabetes mellitus and the function of HPA axis [20,21]. T2DM is associated with increased HPA axis activity and hyperfunction. The negative feedback disappears or weakens, thus the glucocorticoid production increases, and these finally induce the IR and lead to the disorder of glycolipid metabolism [22,23]. One study has reported that the level of 24-h urine free cortisol (UFC) was lower after intensive therapy, suggesting that the functional disorder of HPA axis could be improved after early intensive hypoglycemic therapy in patients with T2DM. We found that the levels of Cor and ACTH in Wuling capsule group and placebo group were significantly decreased. However, the comparison of Cor and ACTH between the two groups was not statistically significant, which might be related to the small sample size and the relatively short-term intervention.

The biological name of Wuling is *Xylaria nigripe*, which belongs to the Xylariaceae family of fungi. Wuling capsules are produced with fungus liquid fermentation technology. Analyzed by HPLC, Wuling capsule contains many bioactive components such as glycosides, adenosine, amino acids (listed in Table 3). The main compounds are 5-hydroxmellenin, 5-carboxylmellein, genistein and 5-methylmellein. It is considered that the effect of Wuling capsule was conducted by a mixture of all components, instead of a single one. The side effects of Wuling capsule have been reported in a Phase IV clinical trial, which including: anoresia (1.3‰), diarrhea (0.999‰), dry mouth (0.666‰), nausea (0.333‰), palpitation (0.333‰), headache (0.333‰), dizzy (0.333‰). These data suggest that it is well tolerated, and most of its side effects are mild.

**Table 3:** Bioactive components of Wuling capsule

No.	Name	Component content%
1	Glycosides	8–12
2	Amino acids	27–32
3	Fatty Acids	8–12
4	Mannitols	8–12
6	Flavonoids	0.1
7	Adenosine	0.08–0.13

## Conclusion

In the present study, we found Wuling capsule ameliorated the depression state of patients with T2DM and depression, and the possible mechanisms might be related to the improvement of inflammatory response and oxidative stress. Although the specific molecular mechanisms were not very clear, these results provide new evidence for the application of Wuling capsule in the treatment of T2DM and its complication depression.

## Abbreviations

ACTH: adreno-cortico-tropic-hormone
Cor: cortisol
HAMD: Hamilton depression scale
HbA1c: glycated hemoglobin A1c
HOMA-IR: homeostasis model assessment of insulin resistance
ISI: insulin sensitive index
SOD: superoxide dismutase
T2DM: Type 2 diabetes mellitus
